# Supplementary material for: Taxifolin Alleviates DSS-Induced Ulcerative Colitis by Acting on Gut Microbiome to Produce Butyric Acid
Source: Nutrients. 2022 Mar 3;14(5):1069. doi: 10.3390/nu14051069 (PMC8912346; doi:10.3390/nu14051069)
Supplement: Supplementary file 1 [file nutrients-14-01069-s001.zip › nutrients-1540159-Supplementary.pdf]

Supplement Table 1. Primer Sequence for quantitative real time PCR.

Gene		5'-3'	bp	Tm	
**FASN**	FASN-F	CTGCTGGACTCGCTCTTTGA	172	60.04	NM_001099930.1
	FASN-R	CTTTGCCTATGTGCTTGCCC		59.83	
**IL-6**	Forward primer	ACAAAGCCAGAGTCCTTCAGAG	75	59.96	NM_001314054.1
	Reverse primer	TGTGACTCCAGCTTATCTCTTGG		59.80	
**IL-10**	Forward primer	ACCTGGTAGAAGTGATGCCC	198	59.09	NM_010548.2
	Reverse primer	TGTAGACACCTTGGTCTTGGA		58.32	
**IL-1β**	Forward primer	TGCCACCTTTTGACAGTGATG	136	59.04	NM_008361.4
	Reverse primer	ATGTGCTGCTGCGAGATTTG		59.55	
**TNF-α**	Forward primer	AGGCACTCCCCCAAAAGATG	208	59.96	NM_001278601.1
	Reverse primer	TGGTGGTTTGTGAGTGTGAGG		60.41	
**P65**	Forward primer	ATTCCGGGCAGTGACGC	185	60.09	NM_001365067.1
	Reverse primer	TCCACATAAGGCCCAGAAGC		59.74	
**GPR41**	Forward primer	TTCCTCCAAGTTCCAAGCCG	83	60.25	NM_001033316.2
	Reverse primer	GGCTGACTTGCTGAGTCCAA		60.25	
**GPR43**	Forward primer	GAATCACAGGAAACGGGAAGCC	176	61.77	NM_001168509.1
	Reverse primer	AGTCTGGGGTCATTCTCCTTG		59.09	
